# Visual Perception of Photographs of Rotated 3D Objects in Goldfish (*Carassius auratus*)

**DOI:** 10.3390/ani12141797

**Published:** 2022-07-13

**Authors:** Jessica J. Wegman, Evan Morrison, Kenneth Tyler Wilcox, Caroline M. DeLong

**Affiliations:** 1 Department of Psychology, College of Liberal Arts, Rochester Institute of Technology 18 Lomb Memorial Dr., Rochester, NY 14623, USA; emorrison@whoi.edu (E.M.); cmdgsh@rit.edu (C.M.D.); 2 Department of Psychology, College of Arts and Letters, University of Notre Dame, 390 Corbett Family Hall, South Bend, IN 46556, USA; kwilcox3@nd.edu

**Keywords:** goldfish, object constancy, object discrimination, picture-object recognition, visual perception

## Abstract

**Simple Summary:**

The ability to visually recognize objects at different viewpoints is known as object constancy and is very important to animals, including humans. By presenting six goldfish with photographs of plastic turtles and frogs at different viewing angles, we were able to better understand object constancy abilities in goldfish. All the fish had successful performance, showing that they were able to distinguish between the turtle and frog photographs, which is evidence of object constancy. This study was the first to look at fish’s ability to identify photographs of objects they had previously seen, known as object-picture recognition.

**Abstract:**

This study examined goldfishes’ ability to recognize photographs of rotated 3D objects. Six goldfish were presented with color photographs of a plastic model turtle and frog at 0° in a two-alternative forced-choice task. Fish were tested with stimuli at 0°, 90°, 180°, and 270° rotated in the picture plane and two depth planes. All six fish performed significantly above chance at all orientations in the three rotation planes tested. There was no significant difference in performance as a function of aspect angle, which supported viewpoint independence. However, fish were significantly faster at 180° than at +/−90°, so there is also evidence for viewpoint-dependent representations. These fish subjects performed worse overall in the current study with 2D color photographs (M = 88.0%) than they did in our previous study with 3D versions of the same turtle and frog stimuli (M = 92.6%), although they performed significantly better than goldfish in our two past studies presented with black and white 2D stimuli (M = 67.6% and 69.0%). The fish may have relied on color as a salient cue. This study was a first attempt at examining picture-object recognition in fish. More work is needed to determine the conditions under which fish succeed at object constancy tasks, as well as whether they are capable of perceiving photographs as representations of real-world objects.

## 1. Introduction

Object constancy is the ability to recognize an object despite changes in the retinal image of the object when it is rotated [1,2]. It is essential to visual perception in humans, with infants beginning to show evidence of these processes at just a few months, or sometimes even days old [1,3,4]. Although very young infants can recognize objects at different orientations, more sophisticated mental representations develop throughout the first several years of life [5]. Animals of most species frequently view their surroundings from different viewpoints. Therefore, possessing object constancy may be advantageous for non-human animals to recognize objects and individuals visually [2,6]. Fish live in aquatic environments, making it easier for them to view objects from a wide variety of orientations, including from above and below. Therefore, it would be especially beneficial for fish to possess object constancy (shape or form constancy) abilities so they are able to recognize objects, such as food sources, predators, or conspecifics, despite changes in the viewing angle [7].

There has been a debate in the literature about how humans recognize objects at different viewpoints that have focused on two theories: viewpoint-dependent representations and viewpoint-independent representations. Many studies supported the idea of viewpoint-dependence [8,9,10] where the visual system encodes the image of an object at the time of viewing, locking in one viewing angle. Multiple 2D representations of the object can then be activated when the object is viewed again in order to achieve object constancy [1,11,12]. Typical performance in this type of study would include decreased accuracy and increased trial time as the stimuli are rotated further away from 0°. However, others found evidence of viewpoint-independent object representations [13] where an object is encoded with a 3D representation that is activated no matter the orientation, size, or location of the object as it is viewed [1,12]. Typical performance in this type of study would include accuracy and trial times that do not differ at all aspect angles of the stimulus. Recent studies have suggested that the human visual system encodes multiple kinds of information that fall in line with both viewpoint-dependent and viewpoint-independent representations, but also information that goes beyond just the orientation of the object such as the textures of the object, or the effects of lighting on an object at different rotations [12,14]. The wide variety of information about an object can then be selectively used by an individual when it is appropriate depending on the recognition task they are involved in [12]. Non-human animals could also benefit from the large amount of visual information about an object by using both viewpoint-independent and viewpoint-dependent processes [15,16,17,18].

To understand if an animal is using viewpoint-independent and/or viewpoint-dependent processes, studies often include stimuli rotated in the picture plane and one or more depth planes. When an object is rotated in the picture plane, the available physical features of the object do not change. For example, imagine you are looking at the driver’s side of a car. If we were to take this image of the side of the car and rotate it in the picture plane by turning it upside down (a 180° rotation), you would see all the same features: the door, two wheels, and the side windows. However, if the car was instead rotated in the depth plane by turning the car to face the backside, different features of the car would come into view (the trunk, the rear window, etc.). Typically, because there are more changes to the available features of an object when it is rotated in the depth plane compared to the picture plane, subjects perform more accurately or faster when stimuli are rotated in the picture plane [9,10,15].

Visual object constancy has been investigated in a wide variety of non-human animals including mice [19], rats [19,20,21], dogs [22], ferrets [23], horses [24], sheep [25], baboons [26], macaques [27], monkeys [28,29,30,31,32], chimpanzees [31], pigeons [6,33,34,35,36,37] chicks [38], bees [39], sea lions [8,40], dolphins [41], and octopuses [21]. Several studies have explored object constancy in fish, such as medaka [42], archerfish [43], cichlids [7], and goldfish [44,45,46,47]. It is important to study a capacity such as object constancy in a wide variety of animals, especially in less-studied species, to better understand an important cognitive and perceptual ability.

Early studies investigating object constancy in goldfish (*Carassius auratus*) utilized black and white 2D stimuli. Most of these studies found evidence that pointed toward viewpoint dependence. For example, when goldfish were trained with 2D black and white simple shapes (horizontal and vertical rectangles) they successfully transferred to stimuli that were rotated up to 30°, but not when stimuli were rotated 40–45° in the picture plane [46]. Sutherland and Bowman [47] trained goldfish to discriminate between 2D black stimuli on a white background consisting of a circle, square, diamond, a square with a knob on top, and a diamond with a knob on top. The fish did not recognize a square or diamond when rotated 45°, but fish performed well when stimuli with knobs were rotated +/− 45°. However, the fish were not successful when these stimuli with knobs were rotated +/− 135°. Bowman and Sutherland [44] found similar viewpoint-dependent results when they trained goldfish to discriminate between a regular 2D black and white square and an irregular square with either a protrusion or an indentation. When the regular square was rotated 45°, the fish did not recognize it. The fish performed well when the irregular squares were rotated 45°, but not when they were rotated 90° or 180°. In contrast, Bowman and Sutherland [48] found preliminary evidence of viewpoint independence. The fish were trained to discriminate between 2D black and white “W” and “V” shapes. The goldfish successfully transferred their training when the shapes were rotated at 90° and 180° in the picture plane, as well as to rectangular stimuli that had the same number of knobs at the top as the letters had points.

DeLong and colleagues [45] further investigated the conditions under which goldfish succeed in object constancy tasks with 2D black and white stimuli. The stimuli consisted of geometric shapes (a half circle and an arrow) as well as line drawings of turtles and frogs, which were not used in earlier studies on goldfish [44,46,47,48]. In a two-alternative forced-choice task, the fish were first trained with the stimuli in an upright position at 0° and later tested with many novel aspect angles (+/−45°, +/−90°, +/−135°, and 180°). Fish successfully discriminated between the stimuli at four of the seven novel aspect angles for both the half circle and arrow (45°, 90°, 180°, and 270°) and line drawings of turtles and frogs (45°, 90°, 225°, and 315°). Overall performance accuracy did not differ significantly between the two types of stimuli. However, the way in which the S+ and S− were presented mattered. The fish showed greater success when the S+ and S− were presented at the same aspect angle (e.g., both at 90°) than when the S− was presented at 0°, and the S+ was presented at a different aspect angle. The results from this study did not clearly point to viewpoint-independence nor viewpoint-dependence. The fish were not able to discriminate between the stimuli at all rotations, but they also did not show a pattern of decreasing performance as the stimuli were rotated further away from the trained viewpoint (0°), which would be consistent with viewpoint-dependence [1]. DeLong and colleague’s [45] study supported and extended prior work with goldfish showing mixed results with 2D black and white stimuli [44,46,47].

Recent studies investigated object constancy abilities in different species of fish using more complex or ecologically relevant stimuli. Newport and colleagues [43] trained six archerfish (*Toxotes chatareus*) to discriminate between frontal views of two different 2D renderings of 3D scanned human faces (both Caucasian women) that were the same color, and all the fish were successful. In a two-alternative forced-choice task, the fish were then shown the faces rotated in the depth plane around the vertical axis up at 30°, 60°, and 90° (a side profile of the face). Across two experiments, all six fish were successful at recognizing the trained faces when they were rotated. However, speed and accuracy decreased as degree of rotation increased, and two fish were not able to recognize their trained S+ at 90°. Therefore, Newport and colleagues [43] suggested that archerfish have viewpoint-dependent representations. Wang and Takeuchi [42] found that female medaka fish (*Oryzias latipes*) were able to successfully discriminate between horizontally flipped (mirrored) male medaka fish faces (using actual male fish as stimuli) as well as non-face 3D inverted objects that were the same color (green or yellow). However, the fish either took more time or failed to recognize vertically inverted faces (180° planar rotation), showing a classical face-inversion effect. The face inversion effect is the phenomena where, when a facial stimulus is inverted, it takes a disproportionately longer amount of time for the face to be recognized compared to other inverted non-face stimuli [49]. Therefore, face recognition in medaka fish could potentially be a different process than recognition of other types of non-face objects, as is thought to be the case in humans and non-human primates, as well as some arthropods [31,32,42,50,51,52].

Schluessel et al. [7] found that Malawi cichlids (*Pseudotropheus* sp.) were able to discriminate between three-dimensional objects rotated in the picture plane and two depth planes. They used stimuli that consisted of small plastic animal models including turtles, frogs, lobsters, and fish in different colors, sizes, and shapes. The stimuli were rotated at 45°, 90°, or 180°, though not systematically (i.e., not all stimuli were presented at each aspect angle). The only rotation where the fish failed to discriminate was when objects were rotated 90° in the depth plane (rotated about the *x*-axis). DeLong et al. [15] replicated and extended Schluessel et al.’s [7] study with goldfish subjects using similar plastic models of green turtles and yellow and red frogs as stimuli. This study was conducted to investigate whether goldfish would perform better using 3D stimuli instead of the 2D black and white stimuli used in prior experiments [44,45,46,47], and perform as well as cichlids. In addition, adding color cues made the stimuli more ecologically relevant and took advantage of tetrachromatic color vision in goldfish [53]. After being trained to discriminate between the turtle and frog at 0°, the goldfish successfully recognized these stimuli at all novel aspect angles (90°, 180°, 270°), suggesting viewpoint independence when the stimuli were rotated in the picture plane. Conversely, fish showed enhanced performance at the trained aspect angle with the two depth plane rotations, suggesting some viewpoint-dependent processes [15].

Goldfish and other fish can show viewpoint independence when viewing 3D color stimuli, especially in picture plane rotations [7,15] but viewpoint-dependence or mixed results when viewing 2D stimuli without color cues [43,45]. To explore whether the goldfish’s success in achieving viewpoint independence is driven by the use of 3D stimuli as opposed to 2D stimuli or the use of stimuli with color cues vs. black and white stimuli, we presented goldfish with 2D stimuli with color cues in the current study. (We also presented goldfish with 3D stimuli lacking color cues in a separate study (C. DeLong, unpublished data)). We used the same fish subjects as in our prior study [15]. We took color photographs of the same plastic model turtles and frogs used in our prior study so that the fish had 2D representations of the 3D stimuli. We also utilized the same experimental set up and procedure as in previous study in which stimuli were rotated in the picture plane and two depth planes. We predicted that the fish would be able to discriminate between the 2D photographs of a turtle and frog as they had done previously with the 3D objects of these stimuli [15], showing that fish can achieve viewpoint independence with either 3D or 2D stimuli so long as color is available as a cue.

Our primary motivation was to continue our investigations of the conditions under which fish exhibit viewpoint-independence vs. viewpoint-dependence. However, because these fish subjects were first trained to discriminate between 3D stimuli then presented with 2D photographs of the same stimuli, this study is also a first attempt at examining object-picture recognition in goldfish. Object-picture recognition is the ability to recognize the correspondence between an actual object and a picture of that object [54]. In the field of comparative cognition, photographs are often presented to non-human animals [55,56,57]. However, it is still unclear for many species if they are able to perceive these pictures as representations of real objects, but not the object itself [56,58]. Pigeons were able to transfer their discrimination of real objects to pictures of those objects [58,59,60,61]. Similar findings have also been discovered in hens [62], bees [63], bears [64], and primates [54,55,57]. In some of these studies, the animal(s) were able to transfer from objects to pictures as well as pictures to objects [58,60,61,62,64]. If the goldfish in the current study could transfer their discrimination from the 3D turtle and frog models to the photographs of turtles and frogs, it may provide partial evidence for object-photo recognition in fish.

## 2. Materials and Methods

### 2.1. Subjects

The subjects of this experiment were six goldfish (*Carassius auratus*), commercially obtained, from 5.0 to 9.5 cm in total length (N = 6). All the fish were approximately 1–2 years of age at the time of data collection. Individual fish were identified by their phenotypic characteristics, including coloration pattern (but sex was not determined phenotypically). All subjects participated in a previous study in which they discriminated between 3D plastic models of a green and gray turtle and yellow and red frog [15]. Of the six fish in the current experiment, four completed all three test blocks in the study. One fish died (Fish 2 during the final test block of the study after completing 17 of 24 trials), and one fish (Fish 1) did not advance to the final test block of the study after failing to meet the training criteria after 38 interim training sessions.

Subjects were housed in 37.85 L tanks (50 cm long × 26 cm wide × 31 cm tall). Each tank contained Top Fin^®^ blue aquarium gravel and an Aqueon^®^ Quiet Flow 10 filter which provided aerated and filtered water. All tanks were additionally aerated by a Top Fin^®^ AIR-2000 Air Pump (PetSmart Inc., Phoenix, AZ, USA). Aqueon water conditioner was added to each tank, which neutralized chlorine and chloramines and detoxified heavy metals, ammonia, and other elements released from fish waste. The lid of each tank contained one Aqueon^®^ 10-watt mini-compact fluorescent light bulb that simulated a 12-h natural light/dark cycle. Each tank was covered on three sides by black cellophane on the exterior walls. Water temperature was between 19 and 24 °C. Water changes (40% of tank) were carried out on a weekly basis and tanks were monitored regularly for pH and waste levels with an API^®^ Freshwater Master Test Kit (Mars Fishcare Inc., Chalfont, PA, USA). Training and testing sessions were conducted once per day, typically five days per week. On days with training or test sessions, subjects were food restricted to prevent overfeeding, which can lead to health issues. On weekdays when the fish were not trained or tested, they were fed twice; once with TetraFin^®^ Golfish Flakes (Tetra GMBH, Melle, Germany) and once with API^®^ Goldfish Premium Pellets (Mars Fishcare Inc., Chalfont, PA, USA). On these days without training or test sessions, the fish were fed at approximately the same time training or testing would have occurred on testing days (in the middle of the daylight cycle).

### 2.2. Stimuli

The stimuli in this study consisted of printed photographs of the 3D stimuli used in DeLong et al. [15]. These 3D stimuli, plastic frog (2.8 cm × 3.4 cm) and turtle (4.0 cm × 2.9 cm) models, were each permanently mounted using Gorilla^®^ super glue gel on a 5.08 cm × 5.08 cm piece of white corrugated plastic, with the frog or turtle rotated at 0°, 90°, 180°, or 270° in the picture plane or depth planes. At the 90° and 270° rotations in both depth planes (about the *x*-axis and *y*-axis), a clear plastic LEGO™ block was attached to the stimulus to allow for better stability and durability when glued to the corrugated plastic. The squares of corrugated plastic to which the stimuli were attached were backed with Velcro^®^ hook and loop strips, which were used to vertically mount the stimuli to a larger stimulus board for training and trials [15]. For the current study, 3D stimuli were vertically mounted in a similar manner, then photographed from a distance of 2.5 cm using an Apple^®^ iPhone SE, with the camera application (Apple Inc., Cupertino, CA, USA). The photographs were taken in the same room in which the testing and training sessions occurred so that the lighting would be similar in the pictures to that of the real 3D objects. The resulting photographs are shown in Table 1.

Images were downloaded and edited using the Apple^®^ Photos application for Macintosh OS X El Capitan (Apple Inc., Cupertino, CA, USA). Images were cropped such that the edges of the corrugated plastic, upon which the turtles and frogs were mounted, were not shown in the image. Stimuli were then printed using a Xerox WorkCentre^®^ 7855 printer on plain Hammermill^®^ white 20 lb. copy paper with dimensions matching the 3D stimuli (5.08 cm by 5.08 cm). The stimuli were then thermally laminated. Stimuli had a border of transparent lamination extending approximately 3 mm beyond the paper print of the stimuli. Two exemplars were made of each stimulus, so that any incidental differences, such as subtle changes to the stimuli that occurred during stimulus creation or during the experiment (for example, subtle damage to the stimuli), could not be used by the fish to reliably discriminate among the stimuli. Each fish’s S+ was the same in the current study as in the previous study [15]. In the previous study, three of the fish were randomly assigned the frog as their S+ (fish 1, 2, 5) and the other three fish (fish 3, 4, 6) were assigned the turtle as their S+ in case there was any reason fish may perform better with one stimulus as the S+ over the other.

### 2.3. Experimental Setup

**Figure 1:**
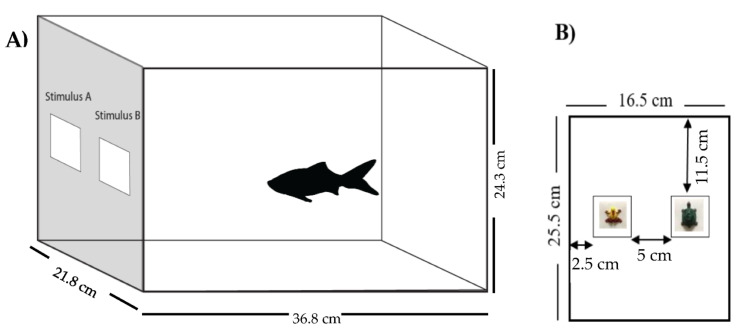
Schematic diagram of the experimental setup. (**A**) Test tank showing the position of the stimulus board and stimuli in the tank. (**B**) The stimulus board containing 0° stimuli (frog and turtle).

Figure 1A shows the experimental setup. During training and testing sessions, subjects were moved to individual 14 L test tanks (36.8 cm long × 21.8 cm wide × 24.3 cm tall). Test tanks contained water and Aqueon water conditioner but contained no gravel. Black cellophane covered all four exterior walls to block external stimuli. The top of the tank contained no lid, allowing easy observation of subjects. To minimize inadvertent cueing, the experimenter was instructed to stand in the front-center of the tank, with their hands and arms at their side, and to not move their entire body (including their head) until the fish made a choice by tapping a stimulus. There were six identical test tanks and fish rotated among the tanks according to a regular schedule, so that the fish were never in the same tank for two consecutive sessions and visited all test tanks once within a six-day cycle. During training and testing sessions, the stimulus board, a white fluted polypropylene (plastic) sheet, was inserted in the front of the tank and rested against the front tank wall (see Figure 1A). On the stimulus board, two stimulus cards could be presented during the training and testing stages (see Figure 1B).

### 2.4. Procedure

**Figure 2:**
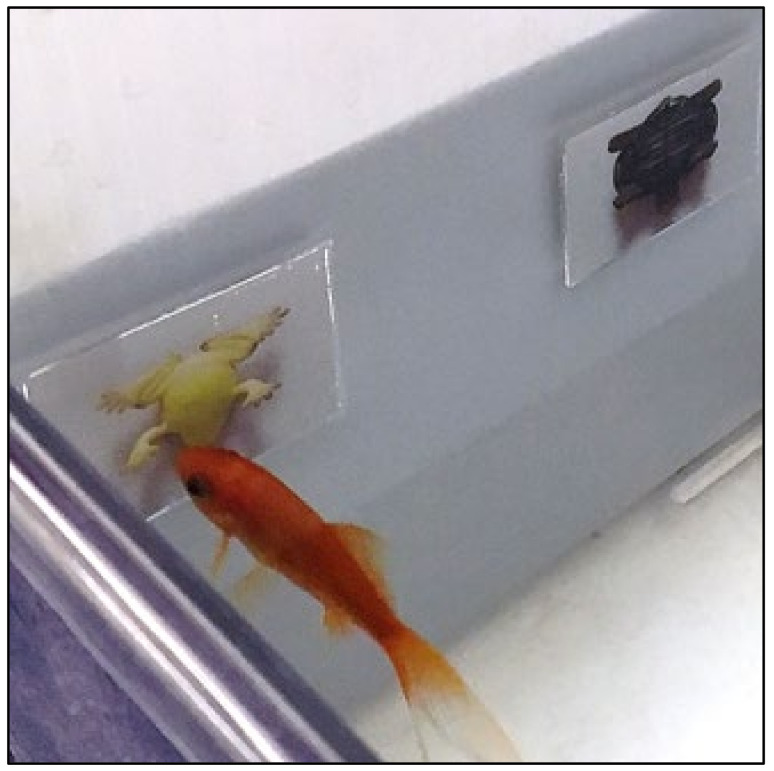
Photo of the experimental setup in the test tank showing a goldfish choosing the frog stimulus rotated 180° in the depth plane (rotated along the *x*-axis) by tapping on it.

Sessions were conducted once per day, with six trials per session, always around the same time of day in the afternoon. There were typically five sessions per week. Each test tank was filled with water approximately equal to the temperature of the home tanks and treated with water conditioner. Each fish was then transferred from its home tank to the test tank with a small net and bowl filled with conditioned water from the test tank and was allowed to acclimate to the test tank for a minimum of 15 min. Stimulus cards were attached to the stimulus board using Velcro^®^, then the fish was led to the back of the tank using a finger dipped just below the surface of the water. This ensured the fish was swimming in the back of the tank and facing away from the front of the tank when the stimulus board was placed in the front of the tank. The placement of the stimulus board and the removal of the experimenter’s finger prompted the fish to swim up to and examine the board, then make a choice by tapping on one of the two stimuli with its mouth (Figure 2).

In the two-alternative forced choice task, the fish were reinforced for correct choices with food paste: a mixture of TetraFin^®^ flakes and water similar to [15,45,65,66]. Food paste was delivered to the fish using a 1.0 mL Luer-Lok^®^ tip syringe (BD, Franklin Lakes, NJ, USA), and about 0.01 mL of food paste was awarded for each correct choice. Experimenter 1 held the syringe out of sight from the fish behind the test tank wall until a correct choice was made. As soon as the subject tapped once on the reinforced stimulus (S+), experimenter 1 lowered the syringe into the tank to the top center section of the S+ stimulus card until the fish ate the food paste. If a fish tapped the incorrect stimulus (S−), it was not reinforced, and the stimulus board was withdrawn. The fish was allowed to choose only one stimulus (S+ or S−) for each trial. The stimulus board was always removed from the test tank during the intertrial interval of about 10–30 s when experimenter 1 recorded the data for the trial. A second experimenter stood out of the line of sight of the test tanks and recorded trial times using a stopwatch (ProCoach RS-013 Water Resistant Sports Stopwatch). Experimenter 2 started the stopwatch when experimenter 1 said “start” as the stimulus board first touched the bottom of the test tank. Experimenter 2 stopped the stopwatch when experimenter 1 said “stop” as soon as the fish tapped the stimulus for the first time. The trial time thus was the time between the board being placed in the tank and the fish making a choice. The fish’s choice (S+ or S−) and the trial time were recorded during the session on datasheet which was used to later enter the data electronically directly from the sheet. Four experimenters trained and tested the fish throughout the experiment. Sessions took approximately 3–18 min per fish (M = 7.3 min for training sessions and M = 7.8 min for testing sessions).

#### 2.4.1. Training

The training phase was brief since the fish already had experience with these stimuli as 3D objects as well as familiarity with the general procedure [15]. We skipped the first two training phases (phase 1, wherein each fish learns to eat from a syringe, and phase 2, where the fish learn to tap the S+ for a food reward) that we have employed in other studies [15,45]. Sessions were conducted using only 0° stimuli, with each trial containing one S+ and one S−. The location of the S+ and S− (left or right) was determined using a modified pseudorandom Gellermann series (67) in which the S+ was never shown more than two trials in a row on the same side to prevent potential side biases. Fish were prompted with the correct answer if they made three incorrect choices in a row. A prompt consisted of presenting the syringe in front of the S+ at the beginning of a trial, so that the fish was guided to select the S+. Prompted trials were not included in the reported choice accuracy for training trials.

Fish were trained for a block of 14 sessions (except fish 1 was trained for 18 sessions due to a side bias) before undergoing the first block of testing to familiarize them with the new stimuli. Fish were also given blocks of at least seven interim training sessions between each block of test sessions to remove biases that may have formed during the previous test block, reduce the influence of each test block on the next, and to ensure the fish remained proficient with the trained task. The fish had to meet two criteria in the training that occurred before the first block of testing as well as the interim training blocks: (1) an average of 75% in overall accuracy (minimum of 63 correct trials out of 84 trials overall for the fish that completed 14 sessions of training, and a minimum of 32 correct trials overall out of 42 trials for interim training) and (2) overall performance accuracy significantly better than chance (according to a summed binomial test).

During both training and testing, if a fish did not choose a stimulus after more than 120 s, the trial was terminated. A short break ranging from 60 s to 240 s was then provided and the trial was repeated. If the fish did not choose a stimulus again after 120 s, the trial and session were terminated, which resulted in missing trials. If a session contained three or more trials in which the fish either did not make a selection or the trial was never attempted due to a terminated session, the session was re-run after the end of the block of 24 sessions, and the results replaced the results of the original day’s session. Data analyses used the chronological order of sessions so that a re-run session was treated as a later session than the original block of 24 sessions (i.e., if the third session of the block of 24 sessions was re-run, then this session was treated as the last session).

#### 2.4.2. Testing

In the test phase, fish continued to be presented with their S+ and S− in a two-alternative forced choice task (the same as in the training phase); however, novel stimuli were added. In each test session, subjects were presented with the 0° stimuli for the first and second trial, then trials 3–6 contained stimuli that were both rotated at the aspect angle of either 0°, 90°, 180°, and 270° (see Table 1). For example, on a 90° trial, both the frog and turtle were rotated 90°. Each of these aspect angles was presented in a random order exactly once each in trials 3–6. Fish were reinforced for correct choices during each trial of testing. There were three blocks of test sessions, with each block containing 24 sessions. All trials within a test block contained stimuli with the same plane of rotation (e.g., in block 1 all stimuli were rotated in the picture plane). All fish received the test blocks in the same order: first the picture plane rotation, then the depth plane rotated about the *y*-axis, and finally the depth plane rotated about the *x*-axis (note that Fish 2 died during the final testing block and Fish 1 did not meet the training criterion to move onto the final testing block). The first two trials of a session always contained exactly one trial with the S+ on the left and one trial with the S+ on the right, and always started on the opposite side from the previous session. For example, if test session 1 trial 1 has the S+ on the right, test session 2 trial 1 had the S+ on the left. As in training, the position of the S+ for each trial was determined by sequences based on a modified pseudorandom Gellermann series [67] with no more than two consecutive appearances of the S+ on the same side and an equal number of trials with the S+ on the left and right.

### 2.5. Data Analyses

Statistical analyses were performed using R v4.1.2 [68]. A statistical significance level of α = 0.05 was used. For post-hoc comparisons, *p*-values were adjusted using Tukey’s WSD [69] method for pairwise comparisons. We modeled testing performance per trial (measured by a choice to the S+ [correct choice] or to the S− [incorrect choice]) using logistic regression fit with the glm() function in R. We modeled testing time (in seconds) per trial needed to make a choice using an inverse Gaussian regression model with a log link function fit with the glm() function in R (for a discussion of the inverse Gaussian and other generalized linear models for modeling psychological response time data, see, e.g., [70]). Multilevel versions of these models incorporating random effects were considered, but none of the models converged using the lme4 R package [71] when variability among fish and/or was modeled with random intercepts—lme4 uses maximum likelihood estimation with adaptive Gauss–Hermite quadrature. This was consistent with results from simulation studies which have demonstrated that convergence rates for multilevel generalized linear models with fewer than 50 subjects or higher-level units (in the case of the current study, fish and sessions) tend to be low. Furthermore, model parameters tend to be underestimated and type I error rates are generally incorrect under such conditions [72]. Instead, fixed effects models where between-subject differences are modeled using fixed effects (subject-specific intercepts and interaction terms) are recommended when the number of subjects is small [73,74,75]. Accordingly, between-fish differences were accounted for using fish-specific fixed effects (intercepts and interaction terms).

For both outcomes, because trials were nested within sessions and sessions were nested within fish, we used within-session and within-fish mean centering for all trial-level variables and within-fish mean centering for all session-level variables [76] in order to disentangle trial-level effects from contextual effects of sessions and fish (i.e., trial-level effects were of primary interest when an experimental factor was manipulated from trial to trial, such as stimuli orientation). Failure to perform such centering with hierarchically structure data can yield uninterpretable effect estimates and inferences [77]. For the same reason, contrasts used to test the effects of experimentally manipulated factors were also centered using this scheme [78]. In addition, change over trials, change over sessions, and an interaction between trial and sessions was modeled to reflect the hierarchical longitudinal sampling design (sometimes referred to as a measurement burst design, [79]). Throughout our analyses, if an interaction was not statistically significant, we proceeded to test the associated simpler effects (i.e., lower-order interactions or main effects) as detailed below. If an interaction was statistically significant, however, we instead used simple (i.e., conditional) effects tests to probe the interaction see, e.g., [80]. Effects were tested using type-III likelihood ratio tests unless otherwise noted. Post-hoc model-based marginal means were computed using the *emmeans* R package [81].

## 3. Results

### 3.1. Training

Of the 528 training trials, the fish completed 519 (98%) trials, so we analyzed the 519 completed trials. Average performance for each fish at the beginning (at 20% of the trials) and end (at 80% of the trials) of training is provided in Table 2 based on marginal mean estimates from a logistic regression model accounting for change over time by including all linear and main effects and two-way and three-way interactions among fish, linear change over trials, and linear change over sessions [79]. The model adjusted for S+ position, starting time per session, tank temperature per session, and two-way interaction effects between fish and each of these three variables. Averaging across fish, the overall performance based on tests of the marginal means on the logit scale from the logistic regression model at both the beginning of training, M = 94.3%, *Z* = 5.47, *p* < 0.001, 95% CI: [85.8%, 97.8%], and at the end of training was significantly better than chance (i.e., 50%), M = 95.3%, *Z* = 3.94, *p* < 0.001, 95% CI: [81.9%, 98.9%]; there was no significant change in average performance across training, *Z* = 0.24, *p* = 0.813.

All six fish began training in the current study 1–10 days after they finished the study with the 3D plastic models of the same stimuli (15). The first time these fish saw the photographs of the turtle and frog on the first trial on the first session of training, all six fish made the correct choice on trial 1. Additionally, three of the six fish (Fish 2, 4, and 5) achieved 100% accuracy in their first session of training. Two other fish (Fish 1 and 3) only made one incorrect choice in their first session, choosing correctly on five out of the six trials.

### 3.2. Testing

#### 3.2.1. Performance Accuracy

Of the 2322 testing trials, the fish completed 2295 (99%) trials, so we analyzed the 2295 completed trials. All trials in each test session were analyzed (including 0°) so we were able to compare performance across all aspect angles. Using a one-sample proportions test, the overall performance was significantly better than chance (i.e., 50%), M = 88.0% (SE = 0.7%), *Z* = 36.39, *p* < 0.001, 95% CI: [86.8%, 99.9%]. Because Fish 1 did not complete the depth (*x*-axis) rotation plane, we could not analyze fish and rotation plane using traditional main effects and an interaction effect as would be the case if Fish 1 had completed all three planes. This also made interaction effects between fish-plane combinations and other variables inestimable. Instead, we included identifiers of the 17 fish-phase combinations in the model and used contrasts to test main effects among fish and among rotation planes.

First, we assessed change over time with respect to trials by session, *χ*^2^(1) = 0.20, *p* = 0.654, sessions, *χ*^2^(1) = 0.17, *p* = 0.678, and trials, *χ*^2^(1) = 0.19, *p* = 0.659. None of the associated likelihood ratio tests were statistically significant for these tests, suggesting that performance did not vary across trials or sessions during testing. There were also no significant differences among stimuli orientations in average performance at the session level, *χ*^2^(3) = 2.64, *p* = 0.450, nor at the trial level, *χ*^2^(3) = 2.45, *p* = 0.484. Fish performed significantly above chance at all four stimuli orientations (see Table 3).

There was a significant interaction between rotation planes and fish, *χ*^2^(16) = 167.89, *p* < 0.001. We then assessed performance among the three rotation planes by using all available fish that completed each rotation plane—Fish 4 had near perfect performance across all three rotation planes and was excluded for computational stability. This suggested that rotation plane did not significantly impact performance, *χ*^2^(2) = 2.34, *p* = 0.310, although performance was significantly better than chance in all three planes: the picture plane (M = 86.7%, SE = 1.3%, 95% CI: [84.2%, 89.2%]), the depth (*y*-axis) plane (M = 84.3%, SE = 1.4%, 95% CI: [81.6%, 87.1%]), and the depth (*x*-axis) plane (M = 84.7%, SE = 1.6%, 95% CI: [81.5%, 87.8%]).

Performance at the trial level was significantly better in trials when the S+ was on the left (M = 91.0%, 95% CI: [88.0%, 93.3%]) compared to the right (M = 87.6%, 95% CI: [83.9%, 90.5%]) between S+ positions, *χ*^2^(1) = 6.90, *p* = 0.009, although the magnitude of the difference was small (3.4%).

There were significant differences in performance among fish, *χ*^2^(5) = 247.02, *p* < 0.001. As shown by the 95% confidence intervals in Table 4, all fish performed significantly better than chance. Fish 5 was the top performer in the test sessions, followed by fish 3, 4, and 6, with fish 1 and 2 at the bottom. The fish showed somewhat similar performance across training and test sessions (see Table 2 and Table 4). In the training sessions, fish 4, 5, and 6 were the top performers, fish 1 and 3 were intermediate, and fish 2 performed the worst (showing a decline in performance). Thus, fish 5 was the highest performing in both training and test sessions, fish 3 was intermediate in training and testing compared to the other fish, and fish 2 was the lowest performer during both training and testing.

Finally, five of the six fish chose the correct stimulus for each test stimulus orientation (0°, 90°, 180°, 270°) in session one of test block one. This was the first time the fish saw the test stimuli (90°, 180°, and 270° stimuli) in the current study. Only Fish 2 made one wrong choice on the first trial of test session 1 with the 0° stimulus but chose the correct stimulus for each of the novel test stimuli (90°, 180°, and 270°).

#### 3.2.2. Trial Time

Of the 2322 testing trials, trial time data were recorded for 1579 trials (68%), so we analyzed the 1579 timed trials. The average trial time was 7.7 s, 95% CI: [7.4, 8.0]. First, we assessed change over time with respect to trials by session, *χ*^2^(1) = 0.72, *p* = 0.397, sessions, *χ*^2^(1) = 1.77, *p* = 0.184, and trials, *χ*^2^(1) = 10.73, *p* = 0.001, suggesting that trial times tended to increase by an average of 3.7% (95% CI: [1.5%, 6.0%]) from the previous trial within a session during testing. There was no significant difference in trial times between S+ positions (left side vs. right side), *χ*^2^(1) = 0.21, *p* = 0.644. Interestingly, trial times were significantly longer when an incorrect choice was made, M = 10.3 s, 95% CI: [9.2, 11.5], than when a correct choice was made, M = 6.7 s, 95% CI: [6.4, 7.0], *Z* = 67.50, *p* < 0.001.

**Figure 3:**
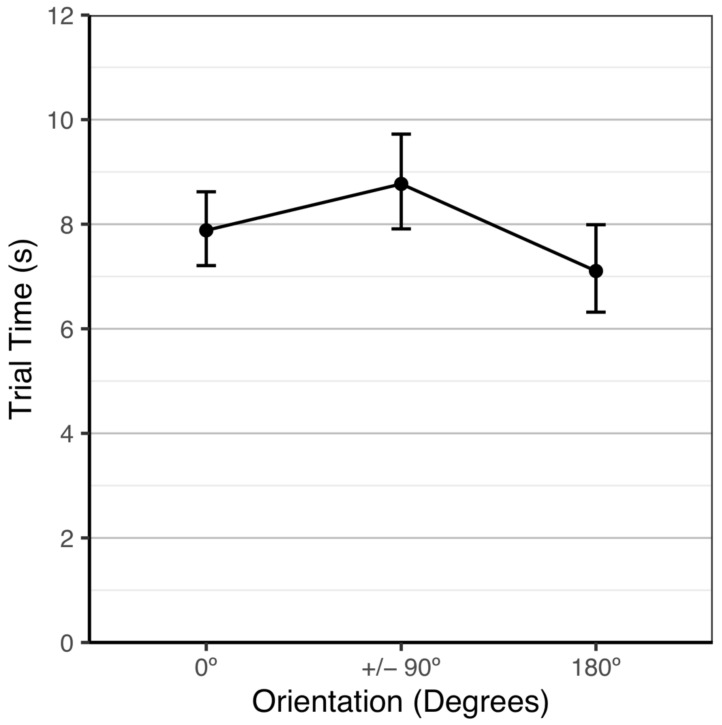
Average testing trial time over stimuli orientations with 95% Tukey-adjusted confidence intervals.

Trial times varied significantly among stimuli orientation, *χ*^2^(3) = 12.91, *p* = 0.005. By examining orthogonal linear and quadratic trends as the stimuli were rotated from 0° to ±90° to 180°, we assessed the functional form of trial time as the stimuli orientation was rotated. While there was no significant linear trend, *Z* = −1.77, *p* = 0.207, there was a significant quadratic trend, *Z* = −3.45, *p* = 0.003, which is shown in Figure 3. The fish were not significantly faster at a 0° orientation (M = 7.9 s, 95% CI: [7.3, 8.5]) than at a ±90° orientation (M = 8.8 s, 95% CI: [8.1, 9.6]), *Z* = −2.16, *p* = 0.079 or at a 180° orientation (M = 7.1 s, 95% CI: [6.4, 7.8]), *Z* = 1.77, *p* = 0.178. The fish were significantly faster at a 180° orientation than at a ±90° orientation, *Z* = −3.57, *p* = 0.001.

There was a significant interaction between the three rotation planes and fish, *χ*^2^(9) = 44.73, *p* < 0.001. As shown in Table 5, there were fish-to-fish differences in trial times among the three blocks. Four fish (fish 1, 2, 3, 6) had faster trial times in the picture plane compared to one of the depth planes, but the other two fish showed a different pattern of results. Fish 2 and 3 had faster trial times for the picture and depth (*y*-axis) planes than for the depth (*x*-axis) plane. Fish 6 also had faster trial times for the picture plane compared to the depth (*x*-axis) plane. Fish 1 had faster trial times for the picture plane compared to the depth (*y*-axis) plane. Fish 4 had the fastest times in the depth (*y*-axis) plane compared to the picture plane and the depth (*x*-axis) plane. Fish 5 had no difference in trial times among the three rotation planes.

#### 3.2.3. Performance Comparison of the Previous and Current Object Constancy Studies

**Figure 4:**
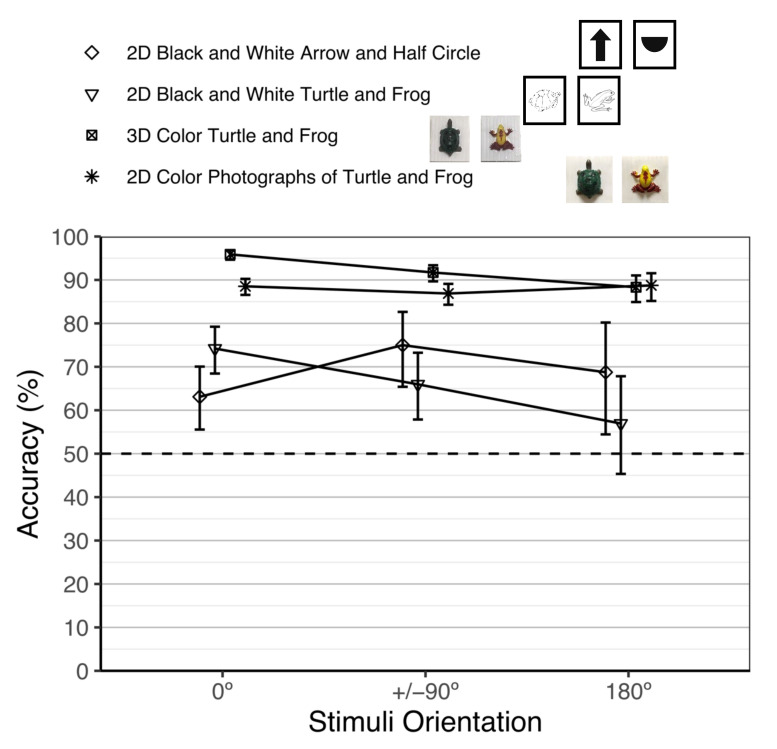
Comparison of performance accuracy on stimuli orientation across our different studies. 95% confidence intervals from the fixed-effects meta-regression model are shown. The 2D black and white stimuli are from DeLong et al. [45], 3D color turtle and frog is from DeLong et al. [15], and 2D color photographs of turtle and frog is from the current study. The fish performed significantly worse at 0° and +/−90° on the current study compared to the 3D color turtle and frog study (there was no significant difference in performance at 180°). The fish performed significantly better on the current study compared to both studies with black and white stimuli.

Figure 4 shows all of our object constancy studies with goldfish. To test for differences in performance between the current study and our previous object constancy studies, we fit a fixed-effects meta-regression model using the *metafor* R package [82] of the average performance accuracy (on the logit scale) and tested the main effect of experiment (2D black and white arrow and half circle, 2D black and white drawings of turtles and frogs, 3D color turtle and frog, current 2D color turtle and frog) while adjusting for stimuli orientation (0°, +/−90°, 180°) and their interaction. There were significant differences in performance among the four experiments, *χ*^2^(3) = 246.39, *p* < 0.001. We then tested for pairwise differences between the current study and the other experiments using Tukey’s [69] correction. Overall performance in the current study (88.0%) was significantly lower than in the 3D study (92.6%), *Z* = −4.90, *p* < 0.001, *Log Odds Ratio* = −0.53, SE = 0.11, 95% CI: [−0.80, −0.25]. Overall performance in the current study (88.0%) was significantly higher than in the 2D black and white drawings of turtles and frogs study (69.0%), *Z* = 10.17, *p* < 0.001, *Log Odds Ratio* = 1.33, SE = 0.13, 95% CI: [1.00, 1.67]. Overall performance in the current study (88.0%) was significantly higher than in the 2D black and white arrow and half circle study (67.6%), *Z* = 7.55, *p* < 0.001, *Log Odds Ratio* = 1.19, *SE* = 0.16, 95% CI: [0.79, 1.60].

To test for differences in performance between the current study and the 3D study at each aspect angle, we fit a fixed-effects meta-regression model of the average performance accuracy (on the logit scale) that included main effects of stimuli orientation (0°, +/−90°, 180°) and Experiment (3D study, current 2D study), and their interaction. The interaction was statistically significant, *χ*^2^(2) = 17.76, *p* < 0.001. We then tested for pairwise differences between Experiments at all stimuli orientations. At 0°, performance was significantly worse in the current study (88.5%) than in the 3D object constancy study (95.9%), *Log Odds Ratio* = −1.11, SE *=* 0.17, *Z* = −6.58, *p* < 0.001, 95% CI: [−1.54, −0.67]. At +/−90°, performance was also significantly worse in the current study (86.9%) than in the 3D object constancy study (91.7%), *Log Odds Ratio* = −0.51, SE = 0.16, *Z* = −3.14, *p* < 0.001, 95% CI: [−0.84, −0.19]. At 180°, however, there was no significant difference in performance between the current study (88.7%) and the 3D object constancy study (88.3%), *Log Odds Ratio* = 0.04, SE = 0.22, *Z* = 0.19, *p* = 0.301, 95% CI: [−0.39, 0.48].

## 4. Discussion

The purpose of this study was to investigate object constancy abilities in goldfish using color photographs of 3D objects that the same subjects viewed in a previous study [15]. During training, fish were presented with stimuli at the 0° aspect angle. During testing, they were presented with the same stimuli at 0°, 90°, 180°, and 270° rotated in the picture plane and two depth planes. Test performance was better than chance at all aspect angles and in all rotation planes, suggesting that the fish were able to successfully discriminate between the stimuli despite changes in orientation. There was no significant difference in performance among the four aspect angles, which suggests viewpoint independence. However, fish had a significantly faster average trial time at 180° compared to +/−90°, which may indicate some viewpoint-dependent processes at work as well. These results do not fit with the usual pattern of results that suggest a mental rotation process where accuracy would decrease and trial time would increase as the stimulus is rotated further away from 0° [8,26,27,83,84,85].

Studies investigating object constancy in both humans and animals have uncovered patterns of performance known as viewpoint-independent (rotational invariance) and viewpoint-dependent (where mental rotation may be used) representations. There has been support for both of these ideas e.g., for viewpoint-independence [6,13,27,34] and for viewpoint-dependence [8,9,10,85] as well as studies that have had findings that did not clearly support either [2,45]. It seems that it is likely both processes can be used depending on the task in humans [12]. The current study seemed to show support for mixed performance in regard to the two theories of object constancy. Taken together with our previous work [15], it seems that goldfish may use viewpoint independent processes when examining performance accuracy results, but support can also be found for viewpoint dependent processes when examining certain trial time results.

Our findings of mixed performance in goldfish do differ from most previous findings on object constancy in goldfish. All but one of these previous studies on object constancy in fish used black and white 2D stimuli [15]. Multiple studies supported viewpoint-dependence in goldfish [44,46,47]. For example, Mackintosh and Sutherland’s [46] goldfish showed good performance with stimuli rotated up to 30°. However, performance fell when stimuli were rotated further away from 0° at 40–45°. An occasional study hints at viewpoint-independence, such as when Bowman and Sutherland’s [48] goldfish successfully transferred their training when the stimuli were rotated at 90° and 180° in the picture plane. This may suggest viewpoint-independence, though fish were tested only tested at two aspect angles in the picture plane. In a more recent study, DeLong et al.’s [45] goldfish (note that none of these goldfish were the same goldfish in the current study) performed significantly better than chance on four of seven novel aspect angles in Experiments 1 and 2, failing to generalize to the other three aspect angles. There was not a systematic decrease in performance as the stimuli were rotated further away from 0° though, so this study also does not provide clear evidence for viewpoint-independent or viewpoint-dependent performance.

Viewpoint dependence has also been supported in other species of fish, including archerfish [43]. Fish were trained with 2D renderings of 3D scanned faces which all six fish could successfully discriminate between. Fish were still successful when the scanned faces were rotated at 30°, 60°, and 90° (except two fish that did not recognize the trained face at 90°); however, accuracy and speed decreased as the stimuli were rotated further away from 0°. This led Newport and colleagues [43] to suggest that the archerfish displayed viewpoint-dependent performance. Wang and Takeuchi [42] interestingly found evidence of the face inversion effect in female medaka fish, using actual male medaka fish as stimuli. The fish were able to recognize the male fish faces when they were horizontally flipped (mirrored) but not when the faces were vertically flipped (180° planar rotation). Therefore, Wang and Takeuchi [42] suggested that facial recognition in medaka fish could be involve different processes than recognition of non-face objects. Since Newport and colleagues [43] used face stimuli, though it was human not fish faces, it could be possible that the archerfish were also using different processes to recognize the stimuli. It is possible that in an object constancy study with non-face stimuli, like the stimuli used in the current study, Newport and colleague’s [43] archerfish may have shown a different pattern of results other than viewpoint dependence.

Two object constancy studies in fish used full color stimuli similar to those in the current study [7,15]. Schluessel et al. [7] presented Malawi cichlids with plastic 3D animal models (such as frogs, turtles, and lobsters) in a two-alternative forced-choice task. The fish were tested with these animal models rotated in the picture and depth plane at 45°, 90°, or 180°. Cichlids were able to successfully discriminate between objects at 45° and 90° in the picture plane, as well as at 45° and 180° in the depth plane, but they failed at 90° in the depth plane. These findings are similar to the current study, though our goldfish were successful at all tested aspect angles, including 90° (0°, +/−90°, and 180° in the picture and two depth planes). This may show that fish are better able to discriminate between rotated objects when they are more ecologically relevant to them by containing color, compared to simple black and white shapes that are not likely to be seen by fish in their natural habitats.

Our previous study [15] utilized the same goldfish subjects (fish 1–6) and the same plastic turtle and frog models. In the previous study, stimuli were presented as 3D objects and in the current study stimuli were photographs of those 3D objects. The fish performed significantly better than chance at all aspect angles (0°, +/−90°, 180°) and in all three rotation planes. There was no difference in performance among aspect angles for the picture plane rotation, suggesting viewpoint-independence. However, fish showed enhanced performance at the familiar viewing angle (0°) in the depth plane rotations. There were individual differences between the fish subjects as there were in the current study. For example, fish 1, 4, 5, and 6 showed no difference in performance as a function of rotation plane, whereas fish 2 and 3 performed best in the picture plane rotation. In addition, the trial times for fish 3, 5, and 6 did not change significantly across the three rotation planes, but fish 1, 2, 4 were faster in the picture plane rotation.

We also saw differences in trial time as a function of rotation plane in the current study. Although performance accuracy was equivalent across the three rotation planes, fish 1, 2, 3 and 6 had faster trial times in the picture plane compared to one of the depth planes (see Table 5). This is consistent with object constancy studies in humans, indicating that such tasks tend to be easier in the picture plane compared to the depth plane [9,10]. In the depth planes when an object is rotated, features that were visible at 0° disappear when rotated, and sometimes new features are added. In humans when such a rotation occurs in the depth plane, reaction time can increase [83,84]. Regardless of rotation plane, fish were always fastest at 180° compared to +/−90°, but not to 0°. These results may indicate that +/−90° was a difficult rotation for the fish, not necessarily that 180° was easy to identify. In the depth plane at +/−90°, more visual information about the shape of the object is lost compared to 180° (see Table 1). For example, at all 180° rotations the four legs that both stimuli have are present, there are limited shadows in the pictures which is similar to 0°, and there are no additional shape elements of the stimuli added that were not seen at 0°). However, at some of the +/−90° rotations, only two of the legs are present, shadows are much larger compared to those in the 0° stimuli, and an additional element of a clear LEGO™ can be seen (this was necessary for the 3D stimuli used in [15] for stability and durability of the stimuli). Therefore, many of the +/−90° rotations could have been more difficult for the fish. They could still accurately discriminate between the stimuli at all the different rotations, as indicated by performance accuracy. However, it took the fish more time to make this discrimination with stimuli at +/− 90.

In both our previous study with 3D turtles and frogs [15] and the current study, performance was above chance for all aspect angles in all rotation planes. This shows that the fish achieved object constancy with both 3D color objects and 2D color photographs of the same stimuli. Additionally, performance in these two studies was higher than performance in our previous object constancy study with 2D black and white simple and complex stimuli [45]. This suggests that goldfish perform significantly better when they have access to color cues. The turtle and frog stimuli used in our current and previous 3D study [15] may have offered the fish important cues, including color and shadows, which may have helped aid the fish in their discrimination of the stimuli. However, it is possible that the fish primarily used color, a 2D non-geometric feature, as a primary cue in both of these studies. Goldfish are tetrachromats, having four cone types [86]. In comparison, humans are trichromats, having three cone types. Humans and goldfish have very similar types of cone photopigments with sensitivities of 450 nm, 530 nm, and 640 nm. At first, it was thought that goldfish only had these same three cone types as humans [87]. However, later studies on goldfish color vision found that goldfish also had a high discrimination ability at 400 nm [86]. Further experiments showed that goldfish have a fourth cone type that is sensitive to ultraviolet (UV) light. Due to tetrachromacy being widespread in nonmammalian vertebrates, it is thought that this type of color vision is likely advantageous for these animals, including goldfish [86]. It is likely that color provides a great deal of information about the visual world for these animals which promotes their survival. Therefore, goldfish may use color as a salient cue both in the wild and when presented with experimental discrimination tasks in a lab. The fish in the current study could have simply relied on color since the stimuli differed in color (a green and grey turtle vs. a yellow and red frog) and these colors were never altered. Therefore, if a fish’s S+ was the turtle and S− was the frog, they could have learned to tap the green/grey stimulus and to avoid the yellow/red stimulus without even considering the orientation of the stimuli. If the fish learned this in our previous study, they may have generalized based on color to the photographs of the stimuli in the current study. Given that there have been many previous studies showing that goldfish are capable of recognizing rotated objects in stimuli without color cues [44,45,46,47,48], we know they can also represent, to some extent, the shape or form of the stimuli. It is possible that the fish in the current study were representing both the color and shape of the stimuli to make the discriminations.

Although performance accuracy was very high across both studies utilizing the 2D and 3D turtle and frog stimuli, the fish had worse overall performance in the current study than the previous study [15]. This primarily reflects worse performance at 0° and +/−90° (there was no significant difference in performance between the two studies at 180°). It may have also been easier for the fish to discriminate between 3D color objects than 2D color photographs, which could account for their difference in performance. The fish may have found it easier to identify features and detect differences among 3D objects than among 2D photographs. Fish typically encounter only 3D objects in their native environment and have no experience viewing photographs (except when being tested in a lab). In our study, photo stimuli were designed to be the same size as the 3D stimuli; however, they may have varied in contrast or saturation, and may have contained additional reflections/specular highlights, due to the lamination. The 3D stimuli may have had higher saturation with more intense colors compared to the photos. This would, in turn, impact the contrast between the stimulus and the white background, potentially making it easier for the fish to see the 3D stimuli better than the 2D stimuli. Additionally, during testing with 3D stimuli, fish could swim around the objects before making a choice, potentially picking up more information from various aspect angles than they could viewing a photograph. That inspection behavior was noted occasionally in other studies [15]. DeLong, unpublished data] but not in the current study.

Another potential interpretation of the pattern of results we found is that the fish may have object-picture recognition where they used their previous experience with a 3D object to recognize it in a 2D photograph. Spetch and Friedman [58] discussed three criteria set out by Ittelson [88] that should be met before concluding an animal perceives correspondence between pictures and objects. These three criteria are for the animal to show transfer from pictures of objects to that object, transfer from the object to pictures of the object, and finally to be able to discriminate between pictures and objects. We found immediate transfer from 3D objects in the previous study [15] to 2D pictures in the current study. All six fish made a correct choice on the first training trial of the first training session. Three of six fish achieved 100% accuracy in their first session, with the other fish only making one wrong choice in their first session. Additionally, there was no difference in performance at the beginning of the training period compared to the end of the training period, showing that the fish were able to recognize these stimuli right away and did not require extensive training to be able to discriminate between the stimuli. We did not test transfer in the opposite direction, from pictures to objects. The third criterion is that the animal can discriminate between pictures and objects. Our finding that fish performed worse with the 2D photos compared with the 3D objects in a previous study could potentially show that the fish noticed a difference between the 2D photographs and the 3D objects. Thus, our study fulfills two of the three criteria for object-picture recognition. Without all three criteria, we cannot make any strong conclusions about whether goldfish perceive a correspondence between pictures and objects.

Past studies examined object-picture recognition by looking at the transfer from objects to pictures as well as pictures to objects [58,60,61,62,64] referred to as bidirectional transfer. In the current study, we only looked at the transfer from real world objects to their pictures since object-picture recognition was not our primary focus. Other studies have only looked at the transfer from objects to pictures as well and did find various species showed recognition of pictures based on previous experience with the object [57,59,63]. However, these did not provide strong evidence of object-picture recognition due to investigating only one direction of transfer between stimuli.

We were unable to find any published studies on object-picture recognition in any species of fish, using either of the two main approaches to study correspondence between pictures and the objects they represent. The first approach is transfer of a learned response between pictures and objects [58]. This was the approach taken in the current study where we presented the fish with the objects first, then used the same task with pictures of those objects. The second approach to studying object-picture recognition is to see if animals respond to pictures the same way they do to natural stimuli that the picture represent [58]. The closest studies we found on object-picture recognition followed this second approach as they examined fish’s responses to mating behaviors through videos of potential mates guppies: [89], sicklebacks: [90]. Both studies showed that fish responded similarly to videos of potential mates as they did to live potential mates. However, these studies used dynamic videos instead of 2D still photographs. Clearly, more work should be carried out to examine object-picture recognition in various fish species.

An interesting question regarding this type of work is whether non-human animals truly perceive pictures as representations of objects, or if they perceive pictures in different ways. Fagot and Parron [56] proposed three possible ways animals may perceive pictures. The first was independence mode, where pictures are processed by animals through the features of the picture itself, independent of any representations of the real object the picture is based on. Another possibility is confusion mode where the animal is simply confused by the picture and perceives it as the same as the object itself. Finally, there is equivalence mode where the animal still associates the picture with the object, but at the same time realizes that the picture is different from the actual object (much like how humans perceive pictures). In order to understand if an animal is in equivalence mode, it would be necessary to meet all three of Ittelson’s [88] criteria described above. In equivalence mode, the picture is seen as a representation of the object, but not the object itself. However, many methods used to understand how animals perceive pictures are limited in that they do not allow for a full understanding of how the animal is processing the pictures. As such, many studies may claim that the animals showed an equivalence between pictures and the real objects, but Fagot and Parron [56] brought up that these studies may not be able to tell the difference between confusion and equivalence with non-human animal perception of pictures.

It is possible that our fish were either in the independence mode or the confusion mode since we met only two of the three criteria for picture-object recognition. With so little known about picture-object recognition in goldfish, the current study offers a first attempt at studying this phenomenon. Future research should attempt to better understand picture-object recognition in goldfish, especially to see if fish are capable of bidirectional transfer between objects and pictures both with color cues and when eliminating color cues as done by Spetch and Friedman [58] with pigeon subjects. In a study looking at object-picture recognition in hens, Railton et al. [62] found that their hens could only transfer between objects and their photographs when color was present as a cue. When color was removed as a cue, the hens (who are also tetrachromats like fish) were not able to transfer between objects and photographs. In the current study, color was always present as a cue, so it will be necessary in future studies to investigate the role that color plays in object-picture recognition.

Future researchers should keep in mind that different fish used different strategies to complete the task in the current study (see Table 4). Although all fish performed significantly better than chance (with a range of 76–99% accuracy), there were still significant differences among individual fish accuracy as well as average trial time (with a range of 4–12 s). For example, Fish 5 had the highest accuracy and was in the middle for trial time, being the third fastest fish. This fish seemed to be able to make her decisions fairly quickly while still being highly accurate in order to gain her food reward quickly and consistently. Fish 3 fell in the middle for accuracy but was the fastest fish. With her average trial time at 4.2 s, fish 3 may have made some wrong choices due to simply choosing too quickly. Fish 2 had lower accuracy (compared to the other fish) and was also the slowest of all the fish. This fish may have not understood the task as well as some of the other fish, leading her to make more mistakes and to take more time choosing a stimulus. It is interesting to investigate these fine-grain differences between the fish which may tell us more about their learning strategy. For example, fish 1 and 2 both had low performance but fish 1 was very fast and fish 2 was the slowest of all the fish. Therefore, speed is not a consistent indicator of accuracy in our study. Schluessel et al. [7] had a similar finding where speed was not a consistent indicator of learning. Their cichlids also showed differences in average trial times (ranging from 0.1 to 2.12 s in experiments 1 and 2). Trial times were compared to the number of sessions to reach the learning criterion, instead of average accuracy as in the current study. However, Schluessel and colleagues [7] also did not find a pattern of trial time and reaching learning criterion. For example, in their first experiment the fastest fish (fish 1) took the greatest number of sessions (16) to reach the learning criterion, while the slowest fish (fish 2) also took a fairly high number of sessions (10). There were other fish who were either slow (fish 8) or fast (fish 7) who took much fewer sessions to reach the learning criteria (3 and 5 sessions, respectively). In addition to variations in accuracy and trial time among fish, individual fish may also utilize cues in the stimuli differently. Some fish may use color as a primary cue in object constancy studies, whereas others can prioritize shape cues when color is unavailable [C. DeLong, unpublished data].

Due to the time requirements of training and testing each individual fish, we were limited to a small sample size of six goldfish, which was in the same range of two to six individuals used in similar studies e.g., [15,43,45,53,91,92,93]. Within-species differences can occur in fish [7,92,94,95] and we did find some individual differences in terms of performance and trial time within our own fish in this study. However, these differences did not impact the overall findings as all fish performed significantly better than chance at all aspect angles in all three rotation planes. Still though, caution should be used when interpreting findings with a small sample size. A power analysis was not conducted a priori; however, the available subjects could not have been increased due to limitations in available aquaria space. We did attempt to compensate for having a small sample size by collecting many trials per subject through a repeated-measures experimental design with 2295 trials (between 299 and 432 trials per fish). Since key experimental variables were manipulated from trial to trial as within-subjects effects, power was thus a function of both the number of subjects as well as the number of trials per subject. As described by Smith and Little [96] when such a design is used, the individuals each become a replication unit. Therefore, by using a repeated-measures experimental design, each subject also served as their own control, which increases power and precision relative to a purely between-subjects design [80,97]. Additionally, this design allows for the investigation into individual differences [96,98] that may not appear if we were to have 100 different fish each complete one trial for each aspect angle. While other independent researchers should also attempt replicate this experiment, having a small sample size may offer some advantages including the ability to closely investigate individual differences and to use each subject as a replication.

There are other limitations to this work beyond sample size. The test trials were only scored during the session by one experimenter. This could allow for influences of experimenter bias. However, it was not ambiguous when the fish would make a choice since they had to physically touch one stimulus that was 6.5 cm away from the second stimulus. However, it may be useful for future research to video record all trials so that a second coder that is blind to the test treatment could score each trial to ensure experimenter bias was not an issue in the study. A second limitation was that all test trials were reinforced, so it was possible that learning was taking place during test sessions, not just during training. However, there was no change in performance during testing across trials or sessions. Therefore, it is unlikely that learning was occurring during the test sessions in this study. In addition, five of six fish performed correctly on the first presentation of each aspect angle (0°, 90°, 180°, 270°) in test session 1 (fish 2 was wrong on the first presentation of the 0° stimuli but correct on the other aspect angles).

## 5. Conclusions

Goldfish discriminated between color photographs of a turtle and frog at 0°, 90°, 180°, and 270° rotated in the picture plane and two depth planes. They performed better than chance at all aspect angles in all three rotation planes. There were no differences in performance as a function of orientation (at 0°, +/−90°, and 180°). However, fish were significantly faster at a 180° than at +/−90°. Taken together with our other work, these results suggest both viewpoint-independent as well as viewpoint-dependent processes. All six fish showed immediate transfer when moving from 3D stimuli in a previous study to the 2D stimuli in the current study, providing partial evidence of picture-object recognition. Since we do not have evidence for bidirectional transfer, when interpreting the results of the current study we cannot assume that the fish see the photographs as true representations of the objects. Fish could have also performed the discrimination in this study as well as in our previous study with 3D stimuli [15] based on color cues. Future research should investigate picture-object representation in goldfish and eliminate color as a cue to see if fish see photos as representations of objects, or if they are just confusing the photos for the objects [17,56]. Additionally, future research should continue to investigate object constancy abilities in fish using more ecologically relevant stimuli [7,15] as this may be more indicative of their natural object constancy abilities. Object constancy could aid fish while navigating throughout their environment by being able to identify conspecifics, predators, and food sources as the swimming fish sees them from various viewpoints.

## Figures and Tables

**Table 1:** Stimuli.

Angle of Rotation	Picture Plane	Depth Plane (*y*-Axis)	Depth Plane (*x*-Axis)
0° *	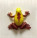 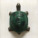		
90°	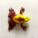 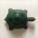	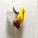 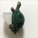	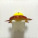 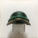
180°	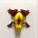 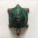	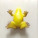 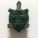	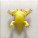 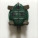
270°	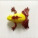 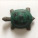	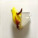 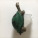	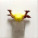 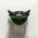

Note. * Only the 0° stimuli were used in the training phase. Testing for all planes of rotation shared the same 0° stimuli.

**Table 2:** Accuracy at the beginning and end of training for all fish.

	Start of Training		End of Training	
Fish	M (%)	95% CI (%)	M (%)	95% CI (%)
1	83.0	[56.7, 94.8]	85.1	[60.7, 95.5]
2	90.6	[69.2, 97.6]	75.1	[53.2, 88.9]
3	81.3	[56.7, 93.5]	88.2	[64.0, 96.9]
4	97.2	[70.4, 99.8]	99.9	-
5	100.0	-	100.0	-
6	99.4	[43.1, 100.0]	96.9	[75.5, 99.7]

Note. Average accuracy and corresponding 95% confidence intervals. Confidence intervals could not be computed for Fish 4 at the end of training and for Fish 5 because performance was near perfect for Fish 4 by the end of training and perfect for Fish 5 in all training trials. For computational stability, the beginning of training was defined at 20% of the trials (trial 16 for Fish 2–6 and trial 21 for Fish 1). The end of training was defined at 80% of the trial (trial 68 for Fish 2–6 and trial 87 for Fish 1).

**Table 3:** Accuracy and trial time in testing for each stimuli orientation.

	Accuracy (%)		Trial Time (s)	
Stimuli Orientation	M	95% CI	M	95% CI
0°	89.9	[85.9, 92.9]	7.9	[7.3, 8.5]
90°	89.5	[83.8, 93.3]	9.0	[8.1, 10.0]
180°	90.2	[84.7, 93.8]	7.1	[6.5, 7.8]
270°	87.4	[81.1, 91.8]	8.1	[7.3, 9.0]

Note. Average testing accuracy and trial time with corresponding unadjusted 95% confidence intervals.

**Table 4:** Accuracy and trial time in testing for all fish.

	Accuracy (%)			Trial Time (s)		
Fish	M	95% CI	Group	M	95% CI	Group
1	76.2	[69.6, 82.9]	A	5.5	[5.0, 6.1]	B
2	81.0	[75.0, 86.9]	AB	11.9	[10.1, 14.1]	D
3	90.2	[86.4, 93.9]	CD	4.2	[3.8, 4.7]	A
4	92.2	[88.8, 95.6]	D	11.3	[9.8, 13.0]	D
5	99.4	[98.4, 99.9]	E	8.9	[7.8,10.2]	C
6	84.5	[80.4, 89.3]	BC	10.0	[8.9, 11.3]	CD

Note. Average testing accuracy and trial time with corresponding 95% confidence interval after adjusting for multiple comparisons with Tukey’s WSD [69]. Fish that share a group did not differ significantly (e.g., accuracy did not differ significantly for Fish 1 and Fish 2, but Fish 3 was significantly more accurate than Fish 1 and Fish 2).

**Table 5:** Trial time in testing for all fish and rotation planes.

Fish	Rotation Plane	M (s)	95% CI	Group
1	Picture	4.2	[3.6, 5.0]	A
	Depth (*y*-axis)	7.2	[6.1, 8.3]	B
	Depth (*x*-axis)	-	-	-
2	Picture	9.4	[7.2, 12.4]	A
	Depth (*y*-axis)	11.1	[8.9, 13.9]	A
	Depth (*x*-axis)	16.1	[12.5, 20.7]	B
3	Picture	4.0	[3.5, 4.6]	A
	Depth (*y*-axis)	4.0	[3.5, 4.5]	A
	Depth (*x*-axis)	4.8	[4.2, 5.5]	B
4	Picture	12.4	[9.9, 15.6]	B
	Depth (*y*-axis)	9.0	[7.6, 10.6]	A
	Depth (*x*-axis)	12.9	[10.5, 16.0]	B
5	Picture	7.8	[6.6, 9.4]	A
	Depth (*y*-axis)	9.2	[7.6, 11.1]	A
	Depth (*x*-axis)	9.8	[8.1, 11.7]	A
6	Picture	8.5	[7.2, 10.0]	A
	Depth (*y*-axis)	10.5	[8.7, 12.6]	AB
	Depth (*x*-axis)	11.3	[9.5, 13.5]	B

Note. Average trial time with corresponding 95% confidence interval after adjusting for multiple comparisons with Tukey’s WSD [69] method within each Fish. Rotation planes that share a group did not differ significantly from each other for a given Fish (e.g., trial times did not differ significantly between the Picture and Depth (*y*-axis) planes for Fish 2, but trial times were significantly longer when the S+ was rotated in the Depth (*x*-axis) plane than the other planes). - = no data.

## Data Availability

The data presented in this study are openly available in the Open Science Framework (OSF) at https://doi.org/10.17605/OSF.IO/5UYGW.
